# Analysis of metabolites in human gut: illuminating the design of gut-targeted drugs

**DOI:** 10.1186/s13321-023-00768-y

**Published:** 2023-10-13

**Authors:** Alberto Gil-Pichardo, Andrés Sánchez-Ruiz, Gonzalo Colmenarejo

**Affiliations:** Biostatistics and Bioinformatics Unit, IMDEA Food, CEI UAM+CSIC, 28049 Madrid, Spain

**Keywords:** Gut-targeted drugs, Gut microbiome, Gut metabolome, New drug modalities, Drug design, Physiochemical properties, Machine learning, Cheminformatics

## Abstract

Gut-targeted drugs provide a new drug modality besides that of oral, systemic molecules, that could tap into the growing knowledge of gut metabolites of bacterial or host origin and their involvement in biological processes and health through their interaction with gut targets (bacterial or host, too). Understanding the properties of gut metabolites can provide guidance for the design of gut-targeted drugs. In the present work we analyze a large set of gut metabolites, both shared with serum or present only in gut, and compare them with oral systemic drugs. We find patterns specific for these two subsets of metabolites that could be used to design drugs targeting the gut. In addition, we develop and openly share a Super Learner model to predict gut permanence, in order to aid in the design of molecules with appropriate profiles to remain in the gut, resulting in molecules with putatively reduced secondary effects and better pharmacokinetics.

**Supplementary Information:**

The online version contains supplementary material available at 10.1186/s13321-023-00768-y.

## Introduction

New knowledge emerging from omics technologies is expanding our understanding of the molecular mechanisms and pathways involved in biological processes. This result in new paradigms for drug discovery requiring new modalities. One of the most important of these paradigms stems from the growing knowledge in the last decade about the crucial role of microbiota on human health. The human body hosts trillions of microbial cells, mainly localized in the gut, that carry a genome (the microbiome) about 100 times the size of the human genome [1–3]. The evidence for the involvement of the gut microbiome in multiple pathologies keeps steadily increasing. This includes areas like obesity, type 2 diabetes, cardiometabolic diseases, non-alcoholic liver disease, diverticulitis, inflammatory bowel disease, colon cancer, etc [4–11]. From this research, a recurrent picture that emerges is that of host-microbiome interactions mechanistically mediated through metabolites in the gut that bind bacterial or human targets [9, 10, 12–17]. In turn, the metabolites can be bacterial, endogenous, or xenobiotics (food, drugs, environmental), or modified versions of any of these produced by putative bacterial and/or host enzymes.

Thus, given all this knowledge, the modulation of all these gut metabolite-target interactions appears as an interesting new drug modality that would tap from the new targets, pathways, and chemotypes appearing from the human microbiome research, as has been suggested [18–20]. This would create new opportunities for treating diseases like the ones mentioned above, plus others like intestinal infectious diseases [21, 22]. Moreover, the ability to modulate the bacterial sub-populations in the gut through new chemicals would pave the way for preventive interventions (instead of curative ones) through novel nutraceutics. This would be an alternative approach to previously used ones based on pro- and pre-biotics to maintain a healthy microbiome. [23, 24]

This new modality could in addition benefit from much reduced distribution and safety issues, as long as the compound is designed to remain in the gut: the administration route would be oral, but with a much more efficient access to the target (it would only require a minimal metabolic stability), and a reduced probability of off-target effects as the compound would not be distributed through the whole body [25, 26]. Alternative approaches to this are based on drug delivery including time-, pH-, and microbiota-dependent delivery systems, and combinations of them [25–27]. In our case we would seek for intrinsic properties of the molecule that make it prone to remain in the gut.

**Fig. 1:**
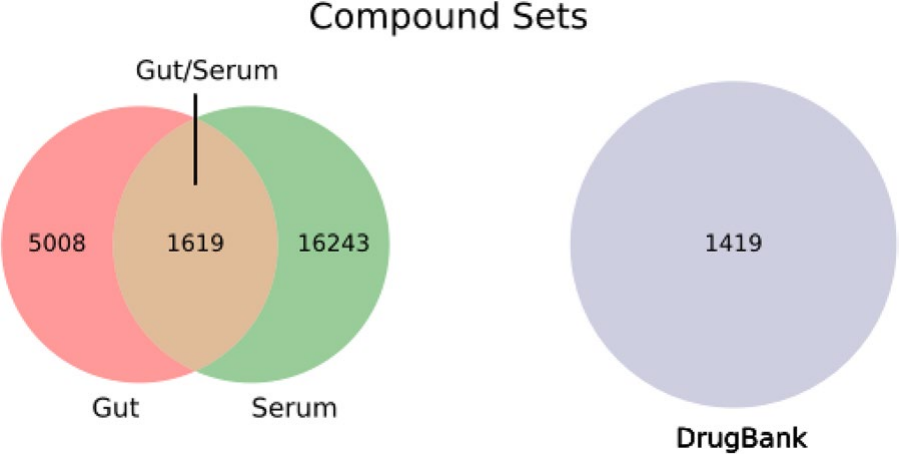
Schema of compound sets used in this work and the corresponding sizes

Taking all this background into account, in the present work our objective is to identify the specific features that gut metabolites have, in order to support the rational design of gut-targeted drugs and nutraceutics. These metabolites are the molecules whose interactions the new drugs would have to modulate. Therefore, the characterization done here provides patterns and features that these drugs will require. This is analogous to the observation that systemic drugs have a greater resemblance to systemic metabolites than to random compounds, which can be rationalized in terms of structural similarity that allows them to compete with endogenous metabolites for their interaction with their targets or with their transporters [28–32].

We analyzed a wide range of structural and physicochemical properties of gut metabolites in comparison with systemic metabolites and drugs, and found significant differences that strongly depended on the chemical class. In addition, in order to predict gut permanence from molecular structures, we tested the use of reversed versions of oral permeability rules like Rule of 5 (Ro5) [33] or Veber’s [34], finding a low predictive power. Thus, we developed a Super Learner [35] model for reliable in silico prediction of gut permanence from molecular structure. This model is available in https://github.com/bbu-imdea/gutmetabos.

## Methods

Data analysis was performed with Python 3.9, and using RDKit [36] 2022.03.2 as cheminformatic toolkit. Metabolite structures and information were retrieved from the Human Metabolome Database (HMDB) [37]; both gut and serum metabolites were retrieved. Only compounds with “detected and quantified” or “detected but not quantified” status were used. Drug structures and information were retrieved from the DrugBank [38], in particular, the subset of small molecules in approved, not-withdrawn, and non-illicit status, ensuring that they acted systemically and were administered orally. Molecular structures were processed and normalized with the ChEMBL Structure Pipeline [39] as described previously [40–42]. A few compounds shared between the DrugBank set and the metabolites sets were assigned to DrugBank. As a result of this retrieval and processing, the compound sets comprised of 5008, 1619, and 1419 molecules, respectively for gut-only metabolites, gut/serum metabolites, and DrugBank sets. A few analyses also considered the set of serum-only metabolites (16,243 molecules).

Ionization class assignment (acid, basic, neutral, and zwitterion) was based on HMDB’ strongest-acidic and strongest-basic pKa’s. Each molecule was assumed to have at least one acidic group if it had a strongest-acidic pKa < 7.4, and at least one basic group if it had a strongest-basic pKa > 7.4. Acid molecules were those with one or more acidic groups and no basic groups; basic molecules were those with one or more basic group and no acid group; neutral molecules were those with neither acidic nor basic groups, and the rest of the molecules were zwitterions. Alternative environment pH values were also analyzed to get an idea of the distribution of ionization classes across different parts of the gut.

The chemotypes of the molecules were analyzed in terms of ClassyFire chemical classes [43]. This is an algorithm and computer program that maps each molecule into a hierarchical taxonomy based on unambiguous, computable structural rules. The taxonomy consists of up to 11 different levels (Kingdom, Superclass, Subclass, etc.) and > 4800 categories.

Tanimoto similarities were based on RDKit path-based fingerprints with default parameters: 2048 bits, 7 bonds as maximum path length.

Bemis-Murcko scaffolds [44, 45] were obtained from RDKit to perform the scaffold analysis. Non-generic scaffolds were used.

For the analysis in the “Other physicochemical properties” section, the following properties were calculated using RDKit (abbreviation within parenthesis): topological polar surface area (tpsa), logarithm of octanol/water partition coefficient (logp), number of rotatable bonds (rb), number of hydrogen bond donors (hbd), number of hydrogen bond acceptors (hba), molecular weight (mw), number of rings (nring), number of aromatic rings (naring), quantitative estimation of drug-likeness [46] (qed), and fraction of sp3-hybridized carbons (fsp3).

Post-hoc analysis of contingency tables was based on adjusted residuals, and cell-specific p-values were calculated with an exact Fisher method recently described [47]. Differences between continuously distributed properties in groups of molecules were tested through a non-parametric Kruskal–Wallis test, followed (when comparing more than 2 classes) by Conover post hoc analysis. The direction of the effect was estimated through the Common-Language Effect Size (CLES) [48] statistic, which estimates the probability than a random observation from one first group would be larger than a random observation from a second group; values > 0.5 correspond to distributions of the first group shifted to larger values, while values < 0.5 correspond to distributions shifted to lower values.

The Super Learner [35] model was implemented in Python using several machine learning base models available in the scikit-learn library. Super Learner is an example of model stacking where a set of base models are used in k-fold cross-validation to generate a matrix of *n* x *m* out-of-fold predictions, *n* being the number of instances and *m* the number of base models. Then, an additional “meta-model” is fitted to this matrix of data to predict the *n* actual outcomes. In parallel, the base models are re-fitted to the complete training data. Once presented with a new external data set, the fitted base models are used to generate the new predictor variables, which are then submitted to the meta-model for prediction. The Super Learner is guaranteed to asymptotically perform better or at least the same as any base model [35]. In our case, we used the following 9 base machine learning models: Logistic Regression, Decision Tree, Support Vector Machine, Gaussian Naïve Bayes, k-Near Neighbors, AdaBoost, Bagging, Random Forest classifier, and Extra Trees. For the final model, logistic regression was fitted. The data was randomly split into 8 folds, keeping the same proportion of chemical classes in each fold, and the first fold was used for external test. The remaining 7 folds were used in the sevenfold cross-validation. As predictor variables, the following physicochemical descriptors were used: tpsa, logp, rb, hbd, hba, mw, nring, naring, qed, and fsp3. In addition, one-hot-encoded ionization class and chemical class were included. This gave a total of 31 predictor variables, that were standardized before use. An alternative deep learning model that used graph embeddings concatenated to the 31 predictor variables provided worse performance, so the Super Learner was finally preferred.

Since, as one reviewer suggested, the use of random splits could overestimate the prediction statistics, we repeated the estimation using non-overlapping, cluster-based train / test splits. In this case, we used Butina clustering [49] to get the clusters as implemented in RDKit, with a similarity threshold of 0.8.

The model and dataset are provided for public use in https://github.com/bbu-imdea/gutmetabos.

## Results

In what follows, we describe an extensive analysis of gut metabolites, in terms of chemical classes, similarity, scaffolds, ionic classes, and a variety of physicochemical properties. For that we will use the set of detected (quantified or not) gut compounds from the Human Metabolome Database (HMDB) [37], corresponding to the feces biospecimen, further processed as described before [40–42] (see also Materials and Methods), which comprises a total of 6627 molecules. In this set of molecules, there is a subset of molecules detected only in the gut (“Gut” set in what follows, 5008 molecules), plus another one of molecules detected in both the gut and serum (“Gut/Serum” set, 1619 molecules).

For comparison purposes, two additional compound sets are included in the analysis: the set of detected (quantified or not) serum metabolites from the HMDB as systemic metabolites (16,243 molecules only detected in serum, “Serum” set), and a set of orally distributed, systemically acting drug molecules obtained from the subset of small molecules in approved, not withdrawn, and non-illicit status of the DrugBank (“DrugBank” set, of 1419 molecules); both additional sets were processed as before [40–42]. Figure 1 displays and schema for all these compound sets, including their sizes and overlap. The idea is to identify physicochemical and structural patterns that are specific for gut metabolites, as compared to serum ones or oral, systemic drugs. We analyzed the distributions of chemical classes, Tanimoto similarity to “DrugBank” set, Bemis-Murcko [44, 45] scaffolds, ionic classes, and physicochemical properties.

Finally, we analyze the problem of gut permanence of molecules, and find specific patterns for molecules remaining in the gut that could be used in the design of drugs acting only locally in the intestine; in addition, a Super Learner model is provided to predict this property from molecular structure.

### Chemical classes of gut metabolites

**Fig. 2:**
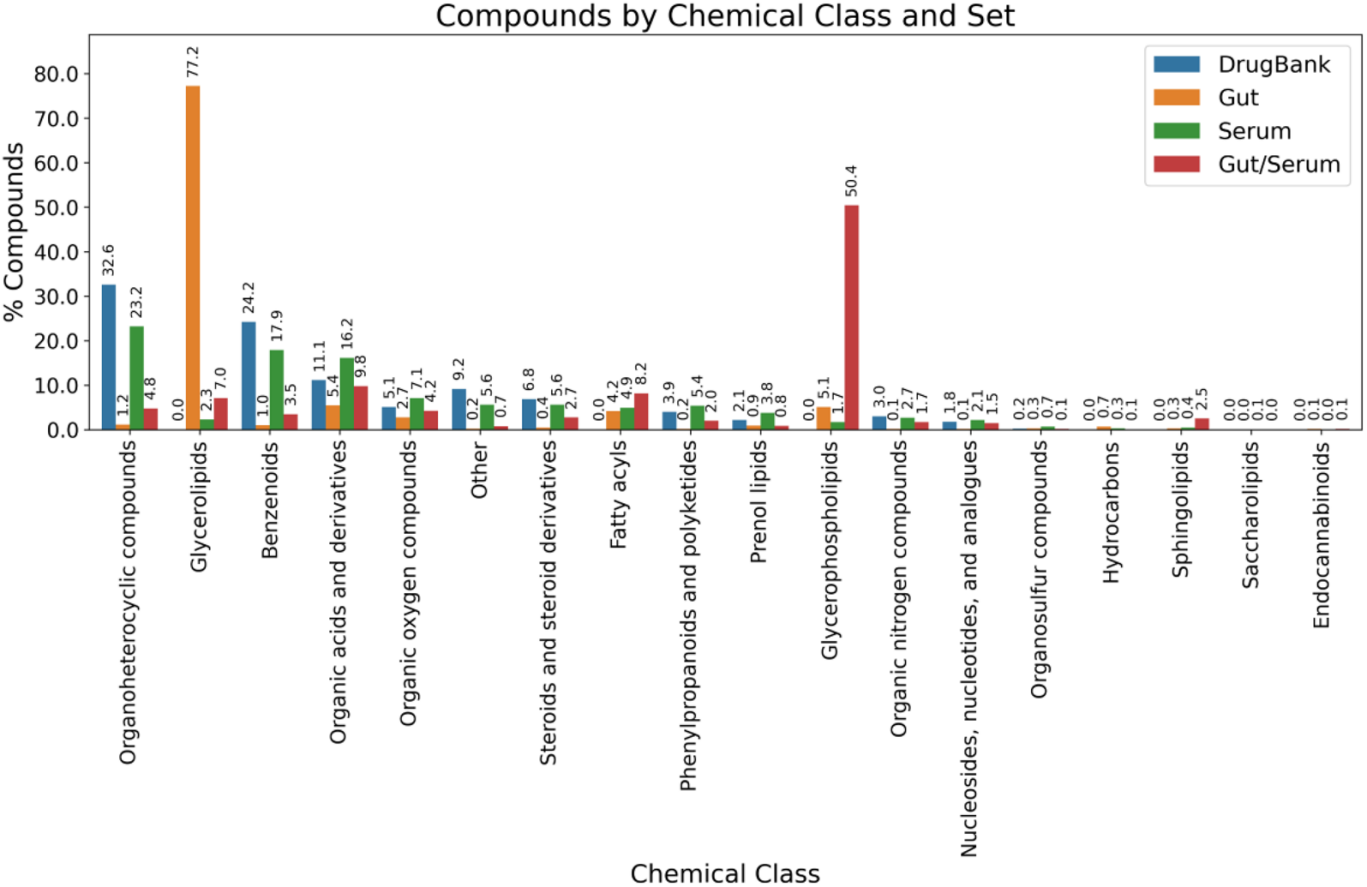
Distribution of chemical classes (based on the ClassyFire taxonomy) for gut-only metabolites (Gut), metabolites shared by gut and serum (Gut/Serum), serum-only metabolites (Serum), and DrugBank molecules (DrugBank)

Figure 2 displays the distribution gut metabolites, for both gut-only molecules (“Gut” class), and those shared with serum (“Gut/Serum”), in 18 chemical classes based on the ClassyFire chemical taxonomy [43]. For comparison purposes, the distributions for serum-only metabolites (“Serum”) and drug molecules (“DrugBank”) are also provided.

These classes are quite diverse from the structural point of view, and include some that are not present in the DrugBank set, like “Glycerolipids”, “Fatty acyls”, “Glycerophospholipids”, “Hydrocarbons”, “Sphingolipids”, “Saccharolipids” (only in “Serum”), and “Endocannabinoids”.

A general inspection allows to see that the distribution of chemical classes in the “Gut” set (5008 molecules) is largely dominated by the over-represented “Glycerolipids” class, that comprises ~ 77% of the molecules. On the other hand, the “Gut/Serum” set (1619 compounds) is dominated by “Glycerophospholipids” (~ 50% of the molecules). The distributions of these two compound sets thus differ considerably from that of “DrugBank” and “Serum” ones, which in turn display remarkable similarities: both have as most populated chemical classes, in the same decreasing order, “Organoheterocyclic compounds” > “Benzenoids” > and “Organic acids and derivatives”; in addition, the six largest chemical classes are the same in both sets, including (besides the three just mentioned), “Organic oxygen compounds”, “Other”, and “Steroids and steroid derivatives”.

Both glycerolipids and glycerophospholipids, together with fatty acyls and sphingolipids, are known for being unable to cross the gut wall. They are hydrolyzed by lipases in the gut lumen in order to be absorbed by the intestine epithelium, where they are again resynthesized and released to the circulation in the form of chylomicrons. Thus, the presence of these compounds in the “Gut/Serum” set (and “Serum” as well) can be ascribed to de novo generation of these compounds and not to permeation through the gut wall. Therefore, in order to better understand the distribution of gut metabolites in chemical classes, we assume that the “Gut/Serum” set would basically correspond to molecules able to cross the gut wall, while “Gut” metabolites would not be able; then, the compounds in the “Glycerolipids”, “Glycerophospholipids”, “Fatty acyls”, and “Sphingolipids” chemical classes within the former set would be reassigned to the later one, reducing the updated “Gut/Serum” down to 516 molecules, and enlarging the “Gut” one to 6111. In turn, we divide the “Gut” set into two subsets: the first one, “Gut-FL”, would include all types of “fatty lipid” (FL) chemical classes, namely “Glycerolipids”, “Glycerophospholipids”, “Fatty acyls”, and “Sphingolipids” (5447 compounds); the second one, “Gut-noFL”, would include the rest of the molecules (664 molecules). This later division would avoid all further analyses of the “Gut” set be obscured by the highly abundant FL molecules, which are quite different from the structural and physicochemical points of view, and show in comparison a much reduced diversity.

**Fig. 3:**
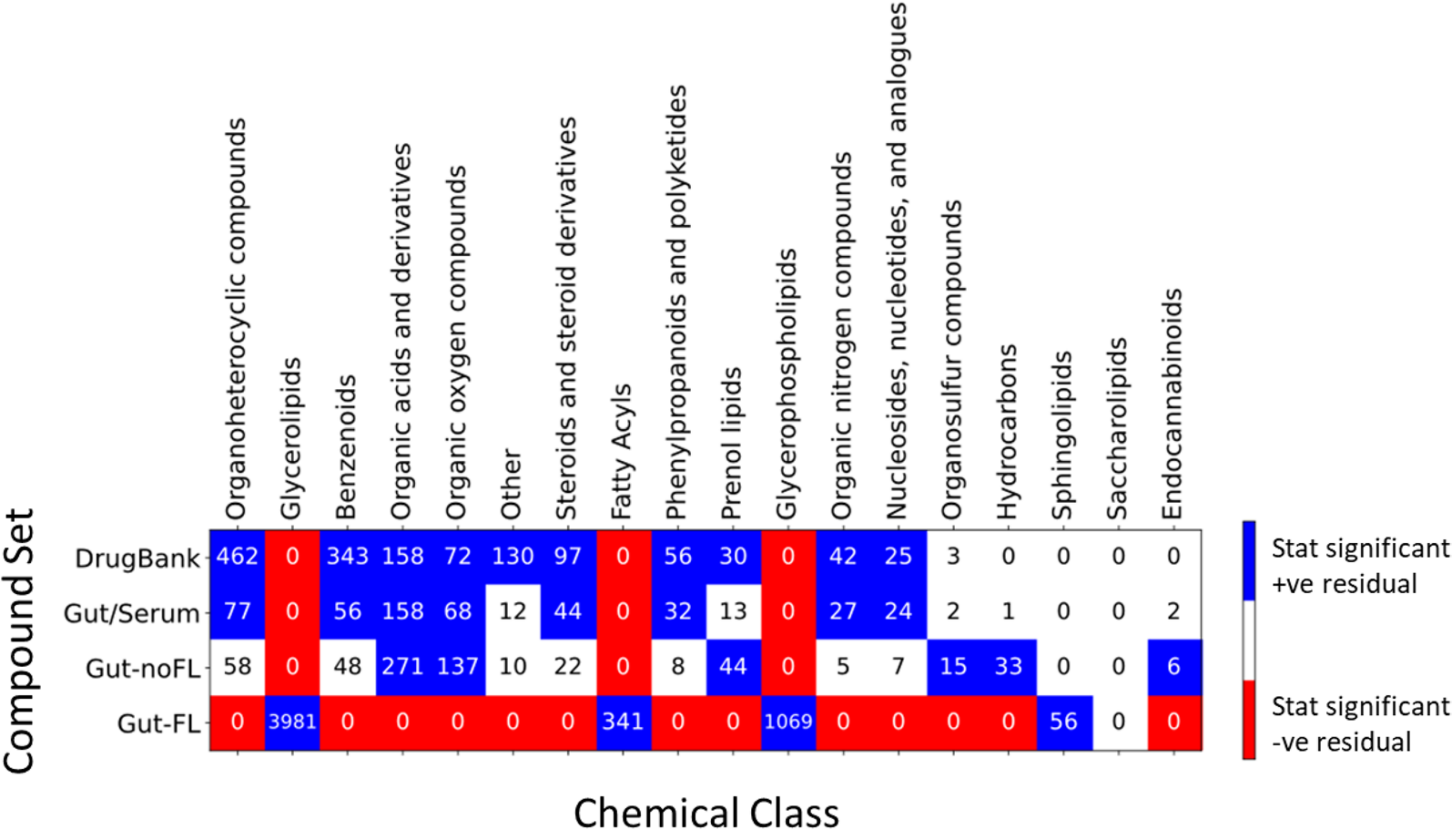
Compound set vs chemical class distributions and enrichments. Adjusted residuals were calculated for the contingency table of compound sets vs chemical classes (cell numbers), followed by a Fisher exact post hoc analysis. Red cells correspond to statistically significant (p-value < 0.05 after Bonferroni correction) under-representation of the compound set vs chemical class, while blue cells correspond to statistically significant over-representation. White cells correspond to not-significant residuals

Figure 3 displays the distribution of compounds across the different chemical classes for these updated gut sets and “DrugBank”, together with the results of statistical tests of the adjusted residuals, in order to better understand over-represented and under-represented chemical classes in the different compound sets.

**Fig. 4:**
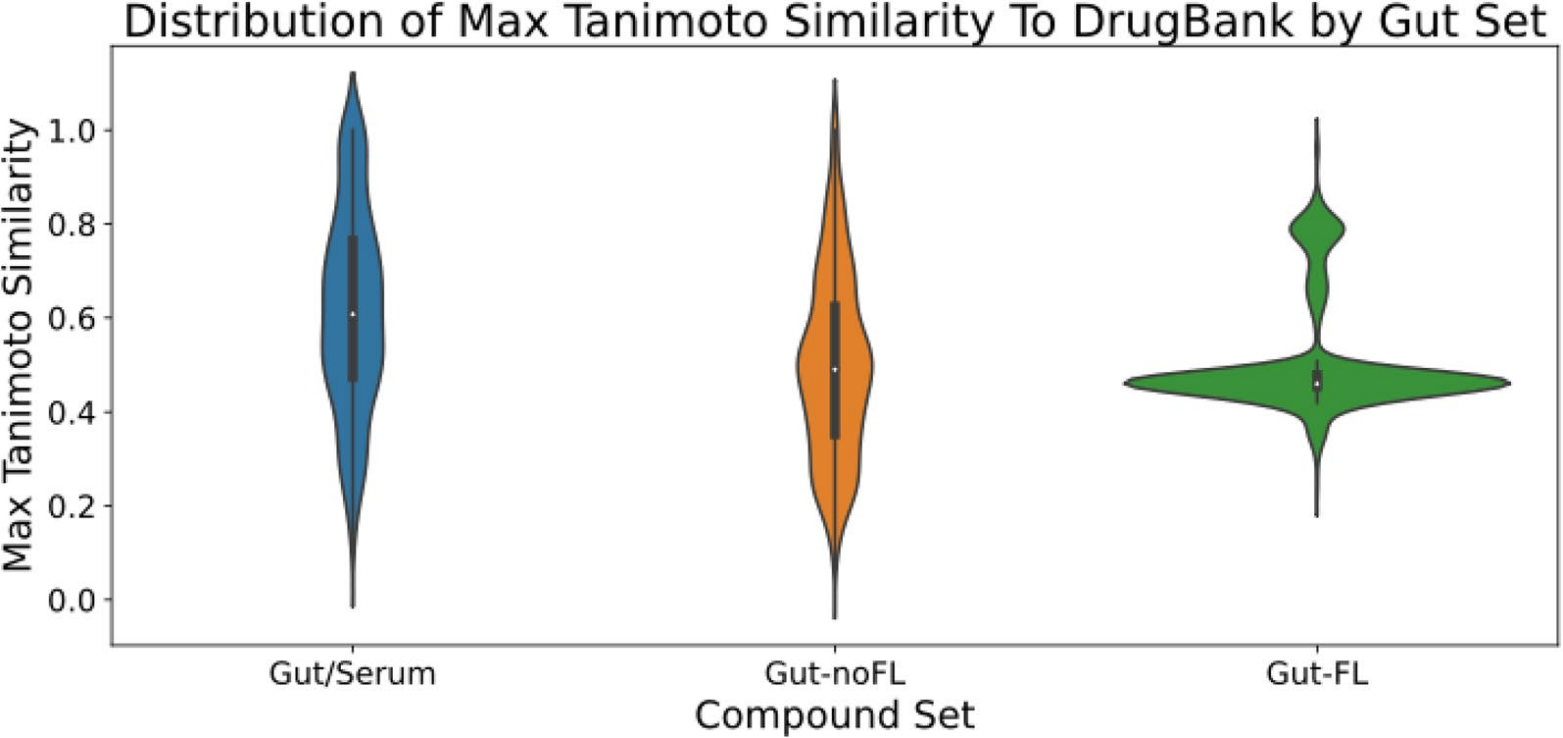
Distributions of maximum Tanimoto similarity of gut compound sets to the “DrugBank” set. For each compound in the gut sets, the maximum Tanimoto similarity observed to any compound in the “DrugBank” set is shown

We can see here a large similarity of the “Gut/Serum” set distribution with that of the “DrugBank” set, having similar over-represented chemical classes: e.g. “Organic acids and derivatives”, “Organoheterocyclic compounds”, “Organic oxygen compounds”, “Benzenoids”, etc. At the same time, the “Gut-noFL” set shows less similarity, with only “Organic acids and derivatives”, “Organic oxygen compounds”, and “Prenol lipids” over-represented as in “DrugBank”, together with “Organosulfur compounds” and “Hydrocarbons”, that are absent or not over-represented in the later set. This would be expected if both the “DrugBank” and “Gut/Serum” sets have chemotypes prone to be readily absorbed by the gut, whether by passive diffusion or through transporters; on the contrary, these chemotypes would be absent in both the “Gut-noFL” and “Gut-FL” sets, that would remain in the gut lumen. As a matter of fact, it is possible to see a higher similarity of the”Gut/Serum” set with the “DrugBank” set in terms of the distributions of maximum Tanimoto similarity to the “DrugBank” set, as can be seen in Fig. 4.

This was confirmed by a statistically significant Kruskal–Wallis test followed by Conover post-hoc analysis, where the pairwise comparisons between “Gut/Serum” and both “Gut-noFL” and “Gut-FL” were statistically significant (p-val < 0.001); in addition, the common-language effect (CLE) statistic was of 0.66 and 0.67 when comparing the “Gut/Serum” distribution vs the “Gut-noFL” and “Gut-FL”, respectively, indicating a shifted distribution towards higher values. The peculiar multimodal density distribution observed for the “Gut-FL” reflects on one hand, the very large number of “Glycerolipids” with little structural variability (high density, low variance component with mode around 0.4), together with two additional modes of low density peaks that correspond to “Glycerophospholipids”, “Fatty acyls”, and “Sphingolipids”.

In the gut sets, the chemical class “Organic acids and derivatives” is basically composed of oligopeptides, short carboxylic acids and derivatives, amino acids and derivatives; “Organic oxygen compounds” comprise sugars, oligosaccharides, alcohols, and ketones; “Organoheterocyclic compounds” include indoles, pyrroles, lactones, etc., and their derivatives; “Benzenoids” comprise derivatives from benzene, benzoic acid, and phenol mainly; “Prenol lipids” include terpenoids, quinones, hydroquinones, etc.; “Steroids and steroid derivatives” collect bile acid derivatives, cholesterol derivatives, etc.; “organic nitrogen compounds” amines and nitriles; and “phenylpropanoids and polyketides” present mainly flavonoids.

The different distribution of chemical classes observed in the gut sets, especially in the “Gut-noFL” and “Gut-FL” ones, to the ones typical of oral drugs, does not preclude their use in drug discovery; instead, they would point towards alternative chemotypes to use for oral drugs when targeted to act locally in the gut in lieu of the typical systemic action. For example, inhibitors like Orlistat (see below), an anti-obesity drug with minimal absorption in the intestine, act in the gut lumen through the inhibition of triglyceride hydrolysis and therefore their intestinal absorption. This drug and other lipase inhibitors act through irreversible competitive inhibition of the lipase catalytic center [50], as they are substrate analogs of glycerolipids. In a similar vein is Acarbose, a substrate analog of the highly abundant oligosaccharides in the gut, that is used to inhibit α-glucosidases and α-amylases in the intestinal lumen, and has negligible bioavailability (see below). These are examples of alternative chemotypes not typical in systemic drugs (analogs of glycerolipids and oligosaccharides, respectively) that have been used to design successful gut-targeted drugs.

### Scaffold analysis of gut metabolites

**Fig. 5:**
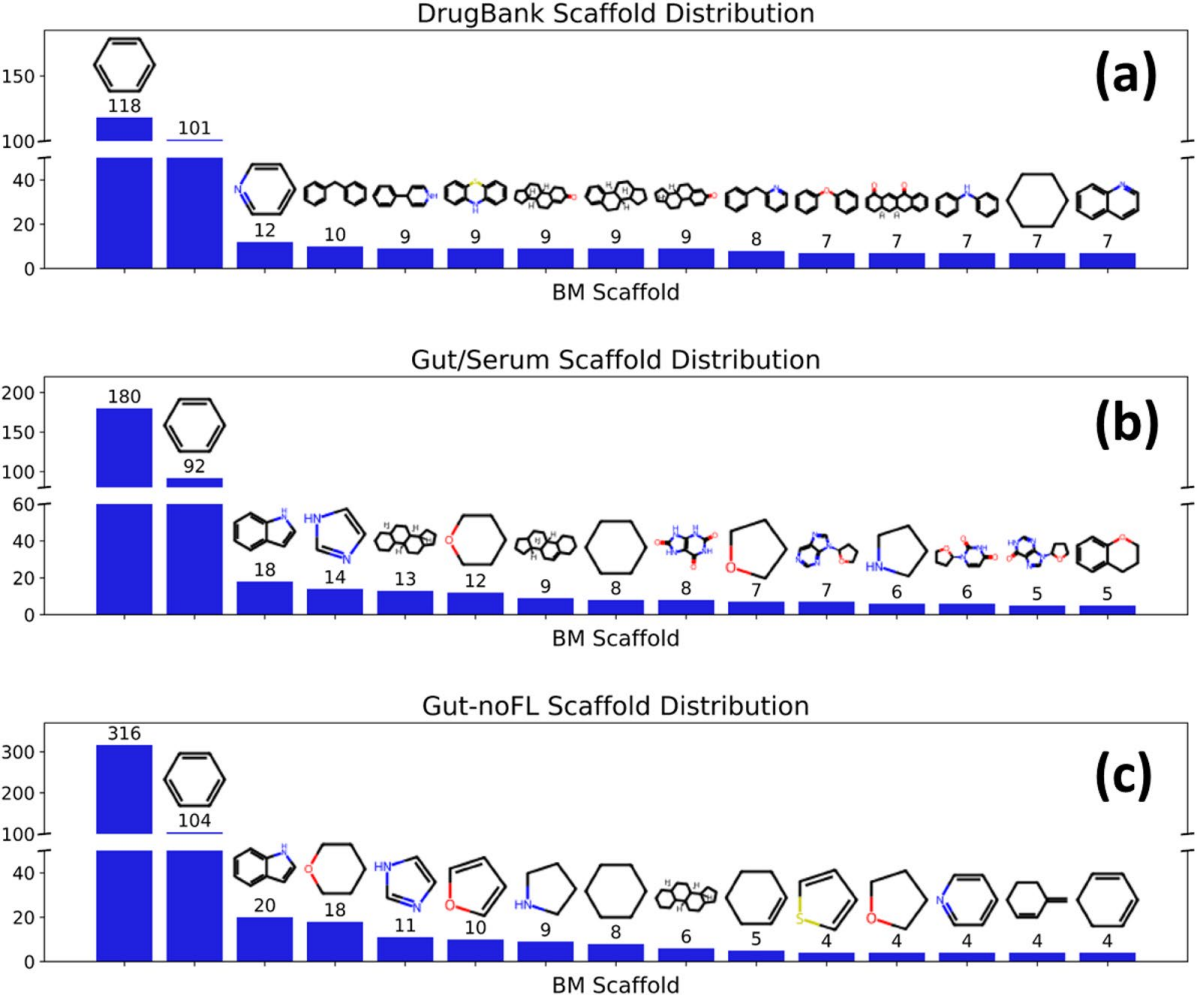
Distributions Bemis-Murcko (BM) scaffols across the different compound sets; top-15 scaffolds for each set are shown. **a** DrugBank; **b** Gut/Serum; **c** Gut-noFL. Gut-FL was not included as it contains a negligible number of scaffolds, in spite of its large size. The bars with no scaffolds correspond to the molecules with no rings, and therefore no BM scaffolds

The structures present in the different compound sets were analyzed in terms of Bemis-Murcko (BM) scaffolds [44, 45], which comprise a summarized representation of a molecule as a set of rings connected by linkers. Table 1 shows the main feature statistics of scaffold distributions in the different compound sets, and Fig. 5 displays the scaffold distributions and structure for the top-15 scaffolds in each compound set. The analysis did not include the “Gut-FL” set as their number of molecules with scaffold was negligible (only 46 out of 5447 molecules).

**Table 1:** Statistics of features of BM scaffolds across different compound sets

Compound set	# mols	# scaffs	Scaff per mol	% mols with scaff	Rings per scaff [avg (SD)]	Arings per scaff [avg(SD)]	Hetrings per scaff [avg(SD)]
DrugBank	1419	874	0.62	92.9	3.54 (1.54)	0.59 (0.32)	0.50 (0.30)
Gut/Serum	516	95	0.18	65.12	2.42 (1.24)	0.41 (0.43)	0.62 (0.43)
Gut-noFL	664	122	0.18	52.41	2.4 (1.41)	0.32 (0.41)	0.55 (0.45)

For each compound set, the number of compounds (# mols), number of unique scaffolds (# scaff), number of unique scaffolds by molecule (scaff per mol), percentage of molecules with scaffold (% mols with scaff), average and standard deviation (SD) of the number of rings per unique scaffold {ring per scaff [avg (SD)]}, average and SD of the fraction of aromatic rings per unique scaffold {arings per scaff [avg (SD)]}, and average and SD of the fraction of heterocyclic rings per unique scaffold {hetrings per scaff [avg(SD)]}, are shown

From this analysis, it can be observed that “DrugBank” is the set with the largest diversity of scaffolds, both in absolute numbers (874 unique scaffolds) and normalized by the set size (0.62 unique scaffold per molecule). Most of these molecules (92.9%) contain scaffolds. In turn, both “Gut/Serum” and “Gut-noFL” have less number of scaffolds (95 and 122, respectively), and of scaffolds per molecule (0.18 in both cases). In addition, their percentage of molecules with scaffold is lower, of 65.12% and 52.41% respectively. Another interesting observation is the larger size of “DrugBank” scaffolds, with an average of 3.54 rings per scaffold, while the two gut sets show averages of about 2.4 rings per scaffold. Moreover, the aromatic content of the scaffolds decrease in the order “DrugBank” (average fraction of aromatic rings of 0.59 in the scaffolds) > “Gut/Serum” (0.41) > “Gut-noFL” (0.32). In turn, the fraction of heterocyclic rings per scaffold is largest in “Gut/Serum” (0.62), but lower in “Gut-noFL” (0.55) and “DrugBank” (0.5).

All these features can be detected in Fig. 5, where the DrugBank scaffolds show larger sizes and more aromatic character, but intermediate heterocyclic content. In turn, the “Gut/Serum” set display smaller rings, with lower aromatic character but higher heterocyclic content. Finally, the “Gut-noFL” set shows smaller rings too, with even lower aromatic character and lower heterocyclic content as well.

### Ionic class analysis

**Fig. 6:**
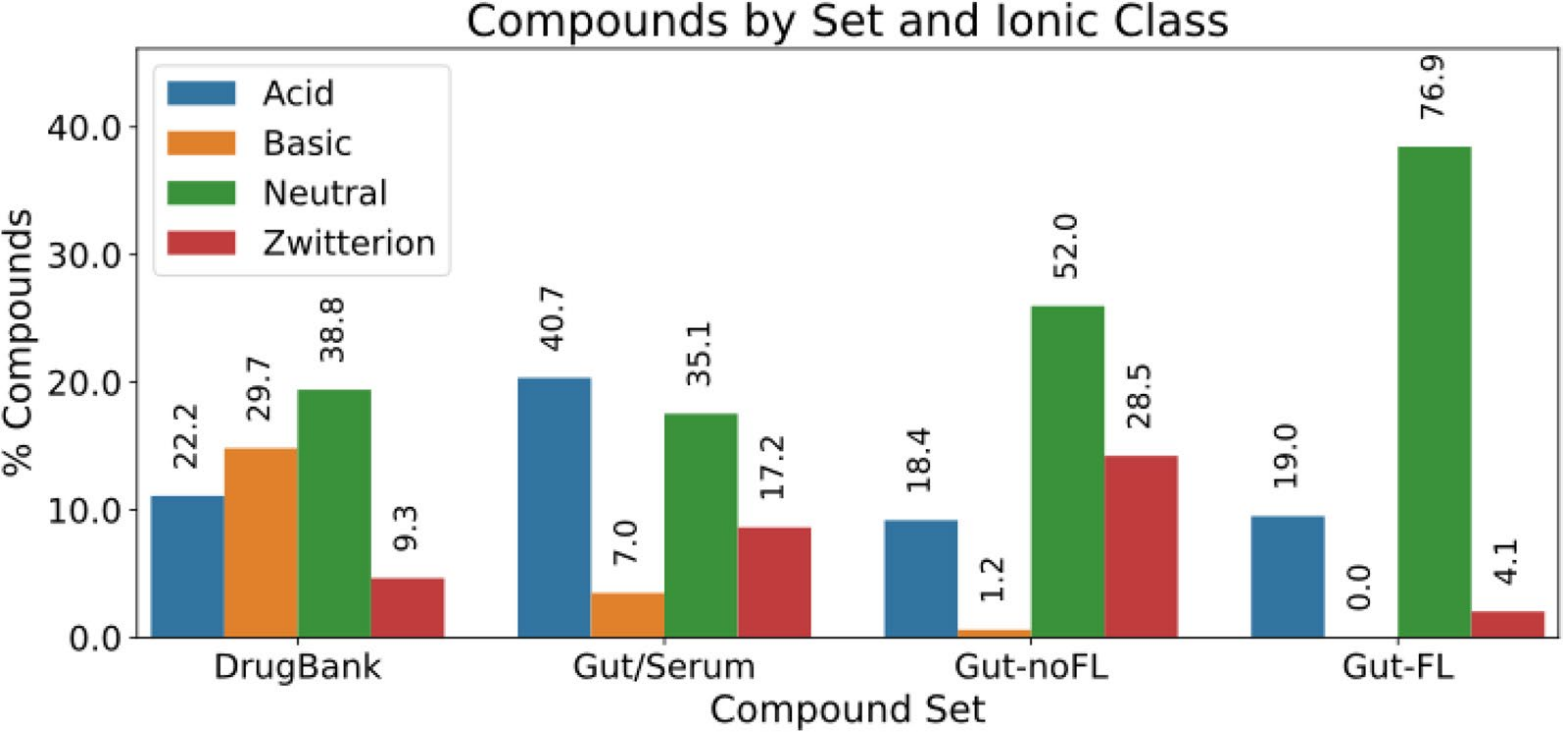
Distribution of ionization states across the four compound sets: DrugBank, and gut metabolites sets

Another interesting aspect to analyze is the comparative ionization behavior of these molecules. Figure 6 shows the distribution of ionization classes (acid, basic, neutral, and zwitterion) in the four compound sets: “DrugBank”, “Gut/Serum”, “Gut-noFL”, and “Gut-FL”.

For this figure, an average pH of 7.4 has been used. Other pH values were also considered in Additional file 1: Figures S1–S3, corresponding to local regions of the intestine: 6.0 (duodenum), 6.4 (caecum), 7.0 (descending colon, jejunum), while 7.4 would mainly correspond to the sigmoid, rectum, and descending colon, plus ileum [26]. We see only modest changes compared to Fig. 6. It is possible to see differences in the ionic class distributions when comparing the “DrugBank” set with the gut sets, and among the three gut sets. In the “DrugBank” set the ionic classes decrease in the order Neutral > Basic > Acid > Zwitterion. However, in the “Gut/Serum” set the acid class is the most abundant one, followed by the neutral class and the zwitterionic class, and the share of basic compounds is the lowest. In the case of the “Gut-noFL” set, there are almost no basic compounds, the neutral class is the most abundant, and in between there are (in decreasing order) zwitterions > acids. The “Gut-FL” set is mainly neutral (~ 77%), with a small share of acids (19%), a very small proportion of zwitterions, and no basic molecules at all.

**Fig. 7:**
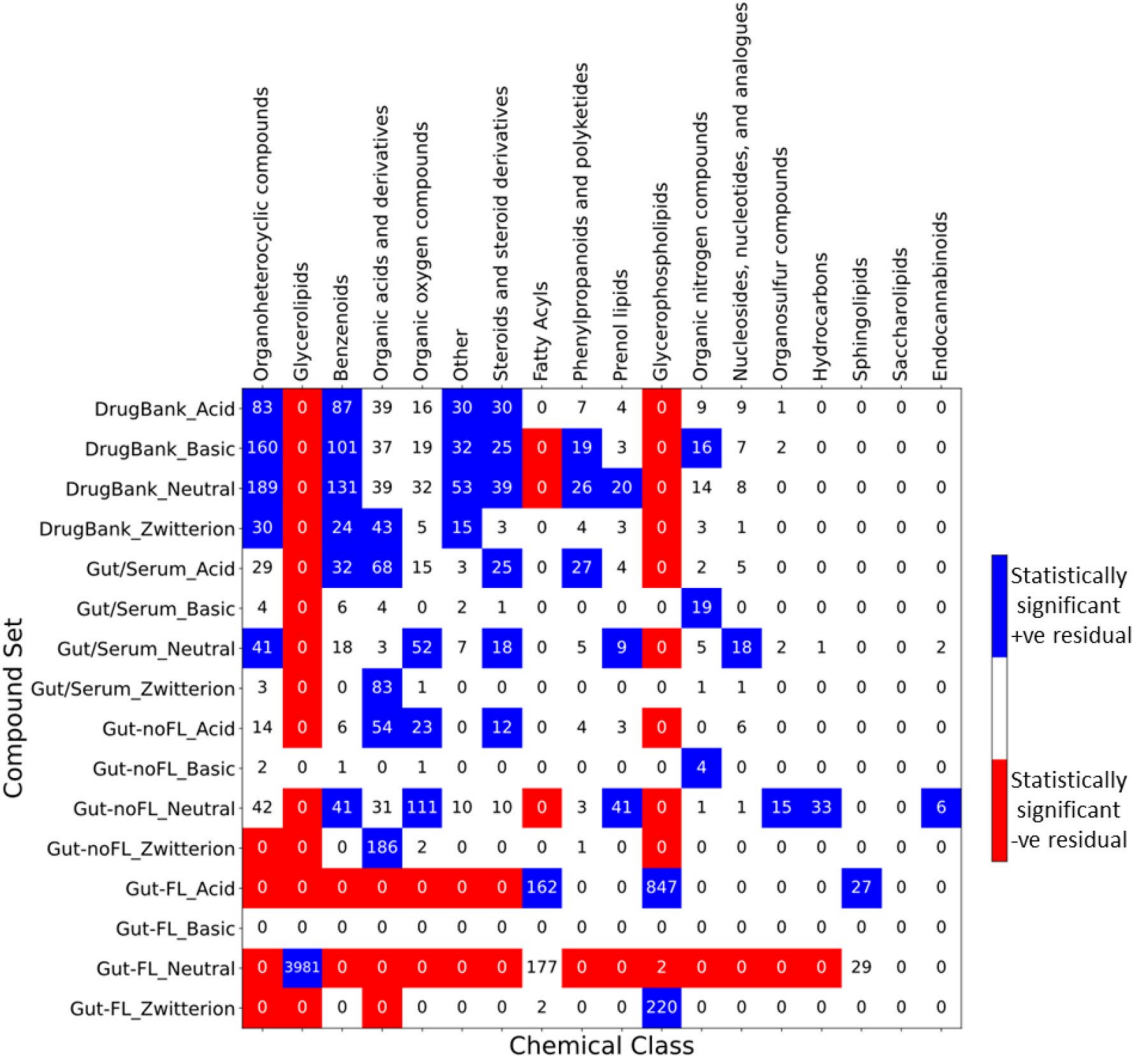
Ionization state enrichment across compound set X ionization classes vs chemical classes. For all the combinations of compound set vs chemical class contingency table, adjusted residuals were calculated, followed by a Fisher exact post hoc analysis. Red cells correspond to significant (p-value < 0.05 after Bonferroni adjustment) under-representation, while blue cells correspond to over-representation. White cells correspond to non-significance

Analyzing the data in terms of chemical classes provide further insights about the observed ionic class distributions. Figure 7 displays the compound set X ionization class vs chemical class contingency table, together with the statistical tests of the adjusted residuals to identify significant over-represented or under-represented combinations.

We see, as expected by design, a significant enrichment of “Glycerolipids” vs “Gut-FL_Neutral”, that is responsible for the large share of neutral compounds in “Gut-FL”. Over-represented cells are also “Glycerophospholipids” vs “Gut-FL_Acid” (major contribution to the acids in “Gut-FL”), “Glycerophospholipids” vs “Gut-FL_Zwitterion” (mainly responsible for the zwitterions), and both “Fatty Acyls” and “Sphingolipids” vs “Gut-FL_Acid” (additional contributions to the acid group).

In the case of the “Gut/Serum” set, the enrichment in acids can be explained by an over-representation of acidic “Benzenoids”, “Organic acids and derivatives”, “Steroids and steroid derivatives”, and “Phenylpropanoids and polyketides” (instead, in “DrugBank”, these chemical classes are predominantly neutral or, in the case of “Organic acids and derivatives”, zwitterions are over-represented). The neutral ionic class is mainly the result of neutral over-represented compounds in chemical classes “Organoheterocyclic compounds”, “Organic oxygen compounds”, “Steroids and steroid derivatives”, “Nucleosides, nucleotides, and analogs”, and “Prenol lipids”; this is largely shared with “DrugBank”, with the exception of “Organic oxygen compounds” and “Nucleosides, nucleotides, and analogs”. Basic compounds result basically from “Organic nitrogen compounds”, and zwitterions from “Organic acids and derivatives”.

Finally, in “Gut-noFL” there are contrasts with both the “Gut/Serum” and “DrugBank” sets. For instance, the neutral compounds, the most populated in this set, are in this case due to an over-representation of “Organic oxygen compounds” and “Prenol lipids” too, but also of “Benzenoids”, “Organosulfur compounds”, “Hydrocarbons” and “Endocannabinoids”, while neutral “Organoheterocyclic compounds”, “Steroids and steroid derivatives”, and “Nucleosides, nucleotides, and analogs” are not over-represented. The acid molecules correspond to “Organic acids and derivatives” and “Steroids and steroid derivatives”, as in “Gut/Serum”, but here acid “Organic oxygen compounds” are over-represented, in addition to the neutral ones. The basic and zwitterionic compounds share sources with “Gut/Serum”: basic molecules are mainly due to over-represented “Organic nitrogen compounds”, and the zwitterions to a very large fraction of over-represented “Organic acids and derivatives”, which in this case more than duplicates that of “Gut/Serum”.

### Other physicochemical properties

**Fig. 8:**
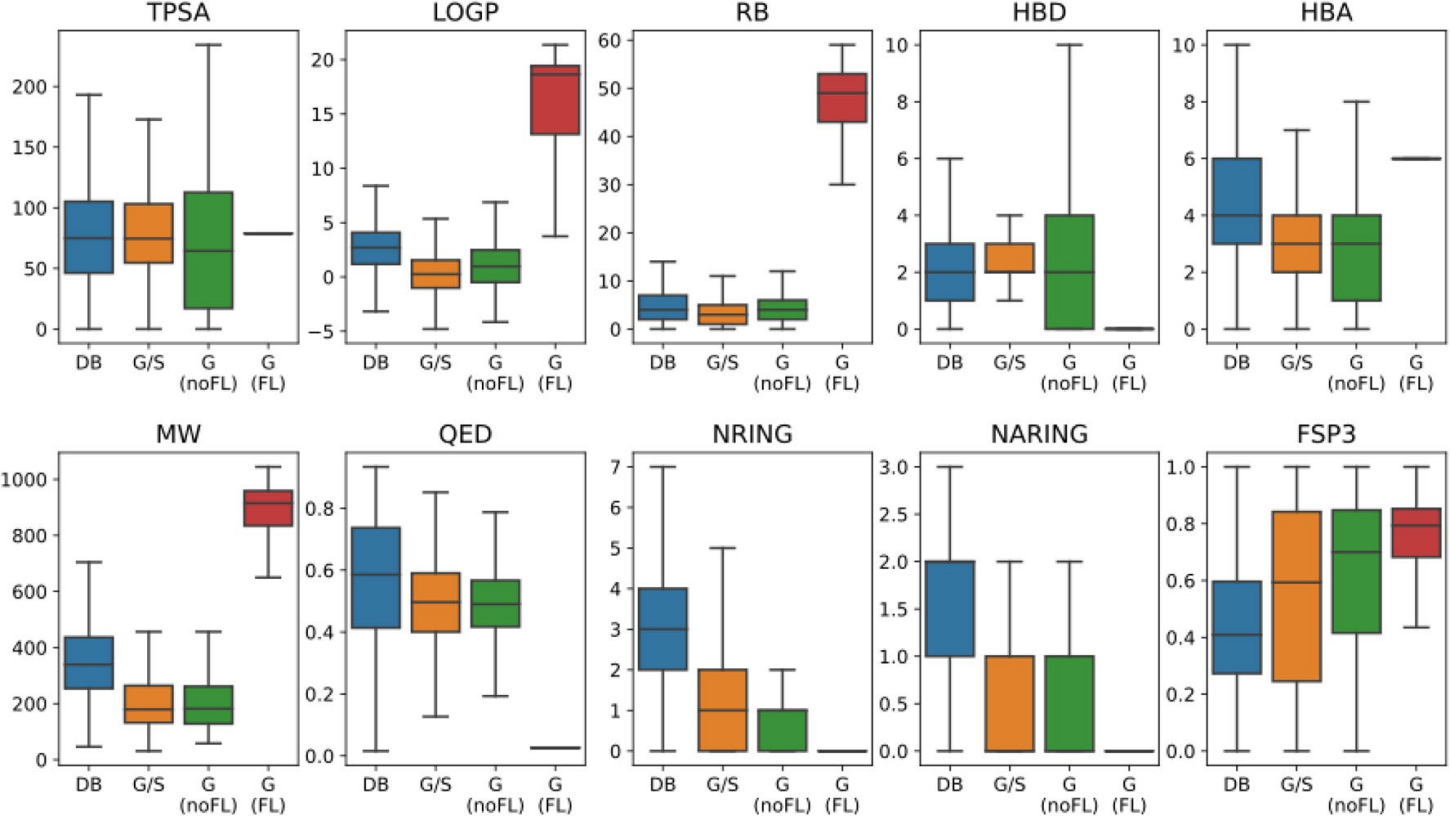
Distribution of multiple physicochemical properties for the different compound sets: DrugBank (DB); Gut/Serum (G/S); Gut-noFL [G(noFL)]; and Gut-FL [G(FL)]. Outliers are not displayed for clarity purposes

To get a more complete idea of additional physicochemical patterns present in gut metabolites, we analyzed a large set of frequently used physicochemical properties, namely: tpsa, logp, rb, hbd, hba, mw, nring, naring, qed, and fsp3 (see Methods for definitions of these abbreviations). Figure 8 displays the distributions of these properties across the different compound sets.

As expected by design, the “Gut-FL” set displays the largest logp, rb, mw, and fsp3 of all the sets, all statistically significant and with CLEs > 0.8 in most of the cases, due to the presence of long aliphatic chains in these molecules. This is accompanied by (almost) no rings, and hbd, and qed basically equaling zero. It is also the group with the largest hba values, with statistically significant CLEs > 0.7 against all of them.

In comparison, the DrugBank set is characterized by lower logp, rb and molecular weight. In addition, it displays the highest qed of all sets (CLEs > 0.6 to the others), and the lowest fsp3 (CLEs < 0.4). All these, not surprisingly, are typical features of molecules compliant with Lipinski rule-of-five, that describe oral, systemic-acting drugs. [51, 52]

In between there are the two other gut sets, “Gut/Serum” and “Gut-noFL”. Compared to “DrugBank”, the most striking features are statistically significant lower logp, hba, mw, qed, nring, naring, and higher hbd, and fsp3. In the case of rb, “Gut-noFL” shows no significant differences with “DrugBank”, while “Gut/Serum” distribution is significantly shifted to lower values. On the other hand, tpsa in “Gut/Serum” shows no significant differences with “DrugBank”, while “Gut-noFL” displays a distribution shifted towards lower values.

### Molecular features associated to in vivo gut permanence

The development of gut-targeted drugs opens the possibility of developing drugs that remains in the gut lumen. In this way, the apparition of side effects and distribution issues could be much reduced, as the body and tissues exposure of the molecule would be constrained to the gut. In addition, lower doses would be required as there would be a much lower dilution of the compound in the gut compartment.

There are a few cases of drugs that act locally in the gut. A collection of them is shown in Table 2.

**Table 2:** Set of gut-acting drugs

Name	Chemical class	Indication	Mode of action	Structure
Acarbose	Organic oxygen compounds	Type 2 diabetes	α-glucosidase and α-amilase inhibitor	
Nystatin	Organic oxygen compounds	Antifugal	Channel-forming ionophore	
Ezetimibe	Organoheterocyclic compounds	Hypercholesterolemia	NPC1L1 cholesterol transporter inhibitor	
Orlistat	Organic acids and derivatives	Obesity	Lipase inhibitor	
Paromomycin	Organic oxygen compounds	Antibiotic, antiamoebic	Ribosome inhibitor	
Kanamycin	Organic oxygen compound	Antibiotic	Ribosome inhibitor	
Neomycin	Organic oxygen compounds	Antibiotic	Ribosome inhibition	
Vancomycin	Organic acids and derivatives	Antibiotic	Peptidoglycan synthesis inhibitor (transpeptidase)	
Mebendazole	Benzenoids	Antihelmintic	Inhibition of tubulin polymerization	
Albendazole	Organoheterocyclic compounds	Antihelmintic	Inhibition of tubulin polymerization	
Pyrantel	Organoheterocyclic compounds	Antihelmintic	Cholinesterase inhibition	
Niclosamide	Benzenoids	Antihelmintic	Uncoupling of oxydative phosphorilation	

Data derived from DrugBank. Drugs were selected if they had a low or null bioavailability, together with a well-defined human or bacterial target (protein or ribonucleoprotein) located in the intestine. Drugs acting through non-specific physicochemical mechanisms (osmotic laxatives, surfactants, ion exchange resins, etc.), or with high bioavailability, were discarded

These molecules have different chemotypes and targets, but all of them have low or null systemic bioavailability. On one hand, we have several aminoglycoside antibiotics that act through inhibition of the bacterial ribosome (Paromomycin, Kanamycin, and Neomycin). Other antibiotic targeting a bacterial target is Vancomycin, a glycopeptide, but in this case the bacterial transpeptidase used for the synthesis of peptidoglycan is inhibited. Several molecules, all of them with heterocyclic structures, have anthelminthic activity, like Mebendazole and Albendazole, which target tubulin polymerization in the worm; Pyrantel, which targets its cholinesterase; and Niclosamide, which uncouples the parasite oxidative phosphorylation. One aminoglycoside compound, Nystatin, is an antifungal agent that acts as a pore-forming ionophore. Finally, there are three drugs acting upon human targets: Acarbose, an oligosaccharide that inhibits pancreatic amylases and gut α-glucosidases; Ezetimibe, an heterocyclic molecule, that inhibits gut NPC1L1 cholesterol transporter; and Orlistat, a triglyceride analog that inhibits gastric and pancreatic lipases. These are used in the treatment of type-2 diabetes, hypercholesterolemia, and obesity, respectively.

From these examples we see that the concept of drugs remaining in the gut lumen has already some exemplars that pave the way for more systematic and extensive drug design efforts, including those coming from novel metabolite-target interactions relevant to disease identified from gut microbiome research.

Intestinal absorption vs permanence is a complex problem, in that some molecules can penetrate the gut epithelium by passive transcellular or paracellular diffusion, while others can through mediated or active transport, and in most cases a mixture of different proportions of these occurs. The molecular features required for diffusion are different from those of mediated or active transport, and therefore a convoluted function of these features would be required to model the whole process for a particular molecule.

This problem can be seen as a reverse-label version of intestinal absorption, which has been thoroughly modeled through the use of in vitro assay data, human or animal pharmacokinetic data, permeation data [34, 53–55], or by analysis of oral, systemic drugs [52, 56]. However, the present dataset can be used to analyze this issue by means of a different endpoint, namely in vivo gut permanence, which is a more appropriate label for our aim, that includes the result of passive diffusion plus mediated or active transport. In addition, it is based on gut metabolites, and therefore provides a better starting point for the design of compounds resembling in vivo relevant molecules. As above stated, it is well known that molecules in the “Gut-FL” set are not able to cross the gut wall [57–59]. In addition, the”Gut-noFL” set can be assumed to comprise molecules not able to cross the gut wall, as none of them has been detected in the serum compartment, and would be putatively excreted in feces unless modified to a permeable form. On the other hand, by definition our “DrugBank” set is made of molecules well absorbed, since all of them are orally administered and act systemically. Finally, the “Gut/Serum” can be approximated to a set of molecules able to cross the gut epithelium too, as they are detected in both gut and serum by definition. Thus, by merging on one side the “DrugBank” set with the “Gut/Serum” set, we would obtain a “Gut-Traverser” set, while by merging the “Gut-noFL” and “Gut-FL” sets, we would achieve a “Gut Lingerer” set. These two sets will form the basis for our analysis.

**Fig. 9:**
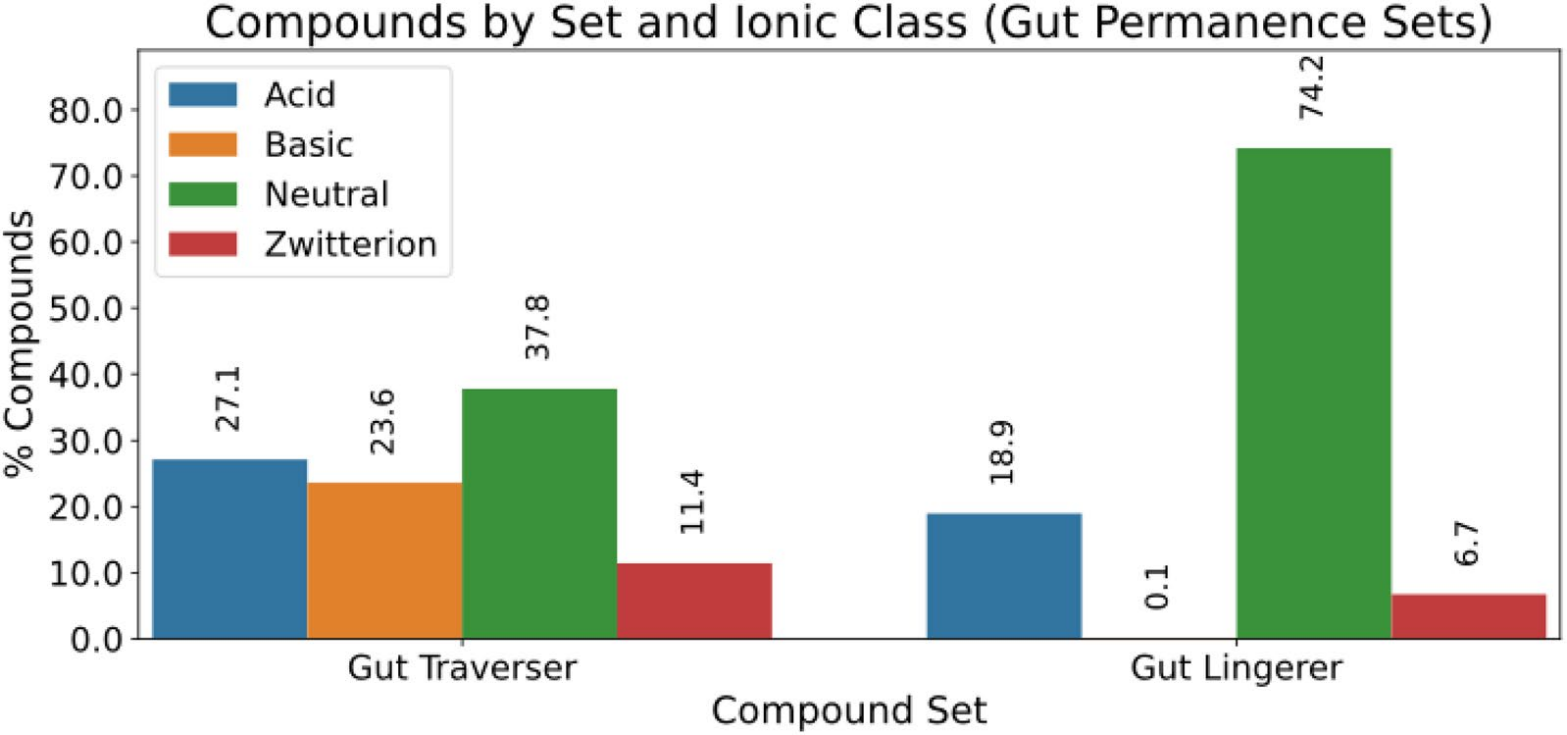
Distribution of ionization states across the two gut permanence sets: Gut Traverser vs Gut Lingerer

Figure 9 compares the distribution of ionization species for the gut permanence sets. An increase of the share in acidic molecules in the “Gut Traverser”, when compared to “DrugBank” is observed, and now the decreasing order of ionization classes is Neutral > Acid > Basic > Zwitterion. On the other hand, the “Gut Lingerers” show an overwhelming majority of neutral molecules (74%), followed by acid ones (18.9%), and zwitterionic ones (67%); basic molecules are almost absent (0.1%).

**Fig. 10:**
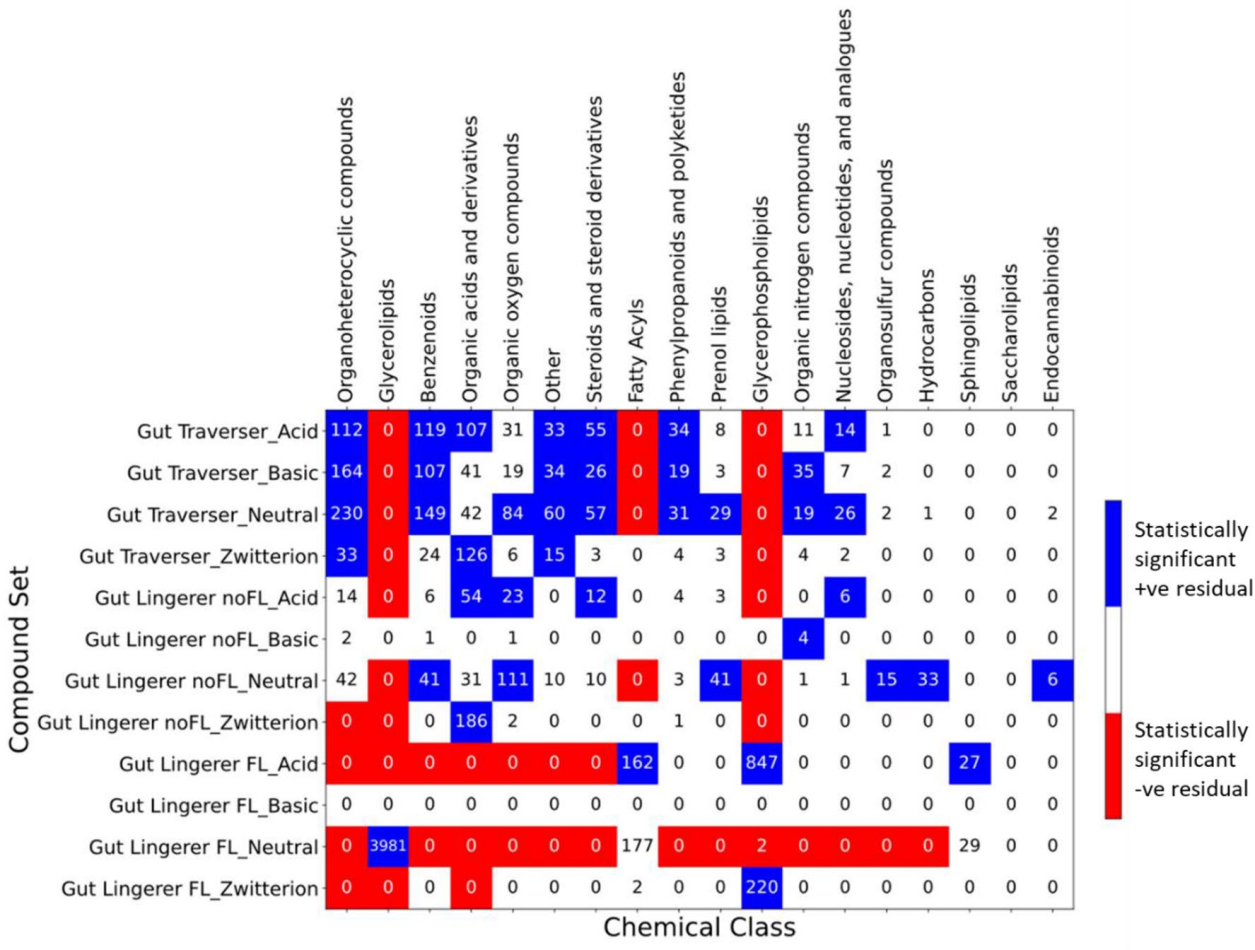
Distribution and statistical enrichment analysis for gut permeation set X ionization class vs chemical class. For all the combinations in the contingency table, adjusted residuals were calculated, followed by a Fisher exact post hoc analysis. Red cells correspond to significant (p-value < 0.05 after Bonferroni adjustment) under-representation, while blue cells correspond to over-representation. White cells correspond to non-significance

In Fig. 10 a further statistical analysis is displayed of the chemical classes vs the gut permanence sets (in this case, “Gut Traverser”, “Gut Lingerer noFL”, and “Gut Lingerer FL”; the latter two corresponding to “Gut-noFL” and “Gut-FL”, respectively, and kept separated here to facilitate the analysis of patterns).

As regarding the “Gut Lingerer” subset, combinations over-represented correspond to neutral “Benzenoids”, “Organic oxygen compounds”, “Prenol lipids”, “Organosulfur compounds”, “Hydrocarbons”, and “Endocannabinoids”; acid “Organic acids and derivatives”, “Organic oxygen compounds”, “Steroids and steroid derivatives”, and “Nucleosides, nucleotides and derivatives”; zwitterionic “Organic acids and derivatives”; and basic “Organic nitrogen compounds”. In the case of the “Gut Lingerer FL” we see the same over-represented classes as “Gut-FL”. Finally, some new over-represented combinations are observed when comparing “Gut Traverser” with “DrugBank”: acidic “Organic acids and derivatives”, “Phenylpropanoids and polyketides”, and “Nucleosides, nucleotides, and analogues”; neutral “Organic oxygen compounds”, “Organic nitrogen compounds”, and “Nucleosides, nucleotides, and analogues”. In addition, zwitterionic “Benzenoids” stop being over-represented.

**Fig. 11:**
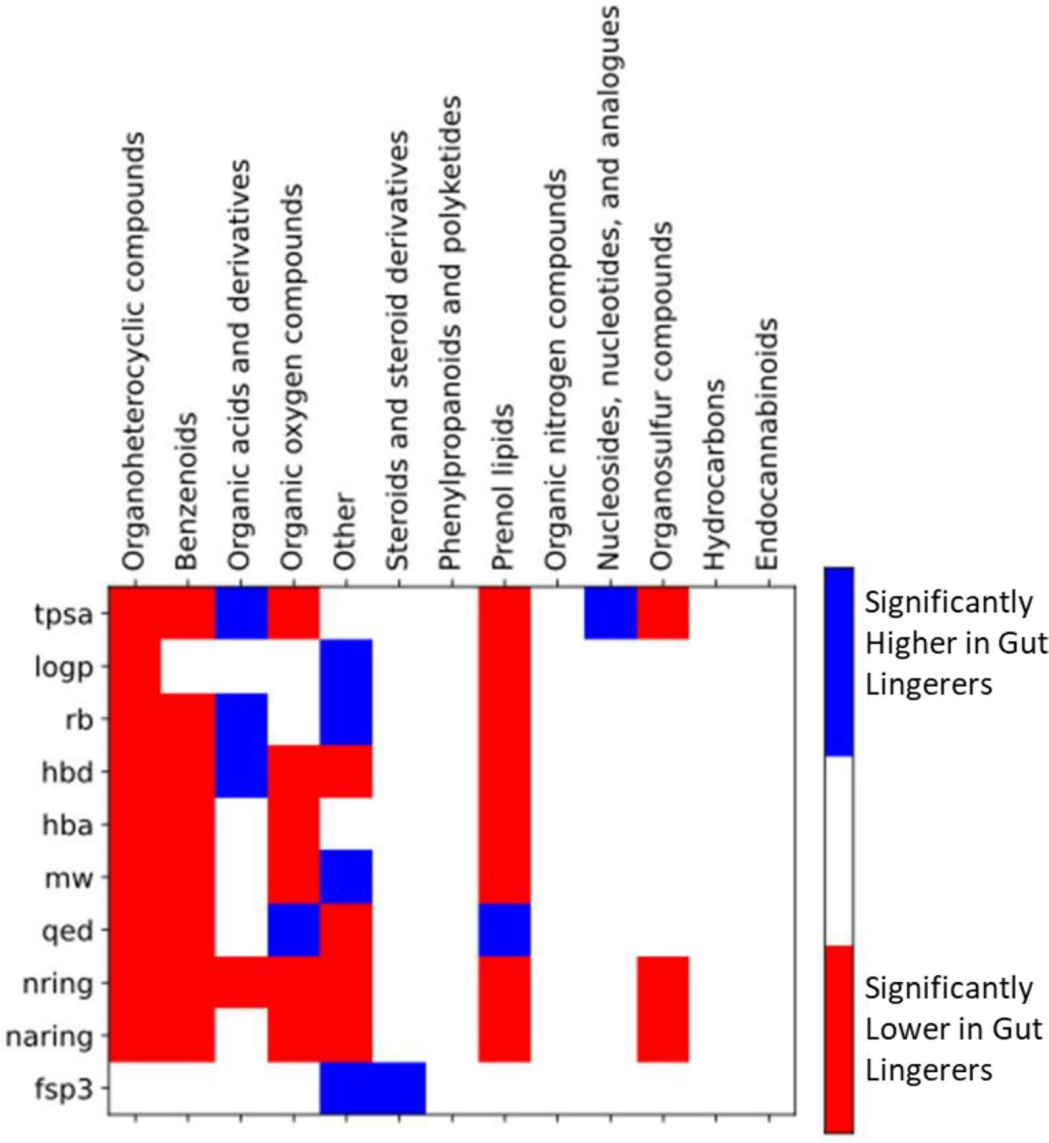
Statistical analysis for the association between different physicochemical properties with gut permeation at the different chemical classes. For all the physicochemical property vs chemical class combination, a non-parametric Mann–Whitney test comparing the distributions in the “Gut Ligerer noFL” set vs the “Gut Traverser” set was performed. Red cells correspond to significant (p-value < 0.05 after Benjamini–Hochberg false discovery rate correction) with a CLES < 0.5, while blue cells correspond to significant test with CLES > 0.5. White cells correspond to non-significance. By reversing the colors we would obtain the significantly higher and lower combinations in “Gut Traversers”. Only shown chemical classes present in both gut permeation sets

Focusing on the set of physicochemical properties above described the profiles for the “Gut-FL” subset have been clarified above: very high logp, rb, hba, mw, and fsp3; and very low hbd, qed, nring and naring. However, for the “Gut-noFL” part of the “Gut Lingerers” it is interesting to further analyze the presence of differential patterns for the remaining chemical classes. Figure 11 shows the statistical analysis of the distributions of the different physicochemical properties in the multiple chemical classes when comparing the “Gut Lingerers” with the “Gut Traversers”.

A variety of statistically significant trends is observed for the different chemical classes. For example, in the case of “Organoheterocyclic compounds”, all the properties but fsp3 are lower in the “Gut Lingerers noFL”. The same pattern is observed for “Benzenoids”, although in this case no significant differences are observed for logp; and “Prenol lipids”, but here qed is significantly higher. “Organic oxygen compounds” have significantly lower tpsa, hbd, hba, mw, nring, and naring, but significantly higher qed. However, “Organic acids and derivatives” show significantly higher tpsa, rb, and hbd in the “Gut Lingerers noFL” set, while nring is significantly lower. The “Other” chemical class displays a mixed pattern, with higher logp, rb, and fsp3, but lower hbd, qed, nring, and naring. “Steroids and steroid derivatives” have significantly higher fsp3, “Nucleosides, nucleotides and derivatives” significantly higher tpsa, while “Organosulfur compounds” have significantly lower tpsa, nring, and nraing.

In terms of properties, we can see that nring, naring, and hba are significantly lower or non-significant for all the chemical classes, while fsp3 is significantly higher in two classes but not significant in the others. The rest of properties show a mixture of trends (higher, lower, non-significant) depending on the chemical classes.

### Prediction of in vivo gut permanence from molecular structure

A machine learning model of Super Learner [35] type was developed to predict gut permanence using this dataset. The dataset was randomly divided into eight stratified folds with equal distribution of chemical classes, and 7 of them were used to perform cross-validation to generate the out-of-fold predictions from 9 base models (Logistic Regression, Decision Tree, Support Vector Machine, Gaussian Naïve Bayes, k-Near Neighbors, AdaBoost, Bagging, Random Forest classifier, and Extra Trees). These out-of-fold predictions were used to train a final “meta-model” (Logistic Regression here) to predict gut permanence in the aggregated 7 folds. Finally, the complete fitted Super Learner model was applied to the 8th fold to evaluate its external predictive power. For a full description of the model, see Materials and Methods. Table 3 collects the predictive statistics of the model: accuracy, precision, recall, F1, area under the receiver operating characteristic curve (AUROC), and area under the precision-recall curve (AUPRC).

**Table 3:** Prediction statistics of model for gut permanence prediction

Pred	Acc	Prec	Rec	F1	AUROC	AUPRC
Ext test	0.96	0.98	0.967	0.974	0.991	0.997
Ext test FL	1	1	1	1	NA	1
Ext test noFL	0.877	0.797	0.702	0.747	0.921	0.833
Ext test stand	0.938	0.899	0.851	0.874	NA	0.916

The statistics accuracy (acc), precision (prec), recall (rec), and F1 (F1), area under the receiving operator characteristic curve (AUROC), and area under the precision-recall curve (AUPRC) are provided for different predictions:, complete external test (ext test); external test for only the “FL” molecules (ext test (FL)); external test for the rest of the fold (ext test (noFL)); and standardized external test (averaging over the two above, ext test stand). Since the “FL” subset comprises only “Gut Lingerer” molecules, it was not possible to obtain an AUROC for it

Since a large fraction of the compounds belong to the “Gut Lingerer FL” subset, with clearly separated features from the rest of the molecules and large structural homogeneity, all of them in the “positive” class, the prediction of this abundant “easy” subset could obscure the predictive power of the model on the rest of the molecules. Thus, in Table 3, in addition to the prediction statistics for the whole external set, the ones for the “FL” and “noFL” subsets are provided, and “standardized” statistics are finally shown as the average of the two subsets, in order to adjust for subset imbalance.

We see that the fit in the case of the “FL” subset is perfect (all applicable statistics equal to one), and remarkably good for the no-FL molecules, with a F1 value of 0.747, an AUROC of 0.921, and an AUPRC of 0.833. The whole model standardized accuracy, precision, recall, and F1 are 0.938, 0.899, 0.851, and 0.874, respectively, with an AUPRC of 0.916. An external prediction based on non-overlapping, cluster-based train / test splits gave similar results (Additional file 1: Table S1), indicating that the prediction statistics are not overestimated due to the use of random splits.

For comparison purposes, the same statistics are shown in Table 4 for both the Lipinski’s [52] and Veber’s [34] rules, reversed to predict gut permanence.

**Table 4:** Prediction statistics of reversed Lipinski’s and Veber’s models to predict gut permanence

Pred	Acc	Prec	Rec	F1	AUROC	AUPRC
Lip ext test	0.862	0.966	0.849	0.903	NA	NA
Lip ext test FL	0.946	1	0.946	0.972	NA	NA
Lip ext test noFL	0.685	0.179	0.06	0.089	NA	NA
Lip ext test stand	0.816	0.59	0.503	0.53	NA	NA
Veb ext test	0.877	0.946	0.889	0.917	NA	NA
Veb ext test FL	0.977	1	0.977	0.988	NA	NA
Veb ext test noFL	0.667	0.278	0.179	0.217	NA	NA
Veb ext test stand	0.822	0.639	0.578	0.602	NA	NA

The same predictive statistics as in Table 3 are shown. No AUROC and AUPRC are provided, as these models do not provide a probability but just a class prediction

The reversed (gut permanence is positive class) Lipinski’s rule is:

Two or more of these:

- mw > 500
- logp > 5
- hba > 10
- hbd > 5

In turn, the reversed Veber’s rule is:

- tpsa > 140, or
- rb > 10

In this case, while the predictions for the “FL” subset are close to perfect (although with a small proportion of false negatives), the prediction for the “noFL” subset is quite poor, with F1 values of 0.089 and 0.217, respectively for Lipinski’s and Veber’s. This indicates that the use of simple rule-based predictions for this problem is not appropriate, especially for the “noFL” part of the gut metabolites. While the “FL” compounds complain perfectly with Lipinski’s large mw, logp, and hba for a compound remaining in the gut, and Veber’s very large rb, the “noFL” subset contains small, low-logp and low-hba compounds that remain in the gut, in contradiction with Linpinski’s rule, as well as moderate tpsa and rb similar to systemic oral drugs, in opposition to Veber’s. Thus, the model here presented appears a more appropriate tool to predict in vivo gut permanence when designing drugs targeted to the gut. We openly share the Python code and dataset in https://github.com/bbu-imdea/gutmetabos.

## Discussion

Gut-targeted drugs and nutraceutics appear as a new drug modality that could exploit the new knowledge coming from the human gut microbiome research. The metabolite-target interactions identified through this research could be modulated by these new drugs and nutraceutics [60], in order to provide novel curative and preventive approaches for health, in multiple areas such as inflammatory bowel disease [9], colon cancer [6, 61], metabolic diseases [5, 62], cardiovascular diseases [11], infectious diseases [21, 22], etc. In addition, the option of directing the design of these compounds to remain in the gut could reduce the distribution, safety, and toxicology problems typical of systemic drugs, the main causes of the high attrition rate in this modality [63].

There are some few examples of drugs acting in the gut and with minimal or null bioavailability. Some of them act over host targets, in the metabolic diseases area; others over bacterial targets, being used as antibiotics; one antifungal, acting as a membrane-pore forming ionophore; and the rest of the molecules, acting on parasitic worm targets, as anthelmintic compounds. In terms of gut microbiome research, so far no commercial drug has been developed based on it, but the use of this research in drug discovery has already been pointed out [18–20, 60], and in fact some initial successful proof-of-concepts have allowed to find inhibitors of the pregnane X receptor based on gut metabolite mimics [64]. This has been followed by the development of inhibitors of the aryl hydrocarbon receptor, based on metabolite mimics too [65, 66]. In addition, in other work a combined bioinformatic/cheminformatic analysis based on data from the Human Microbiome Project has allowed to suggest several target-metabolite interactions that could be useful in drug discovery for inflammatory bowel disease [67].

Given all this background, the current work provides useful analyses that will help in the rational design of gut-targeted drugs based on (host or microbial) gut metabolites. This work has identified two subsets of gut metabolites: those present only in the gut (“Gut” subset), and those also present in serum (“Gut/Serum” subset). In turn, the former can be split in two additional subsets, a very large one with “FL” type of molecules, that is, molecules in the “Glycerolipids”, “Glycerophospholipids”, “Sphingolipids”, and “Fatty acyls” chemical classes (“Gut-FL” subset), and another one including the molecules with alternative chemical classes (“Gut-noFL”). From this analysis it has been possible to identify general physicochemical and structural patterns in the gut sets that differentiate them to the set of oral, systemic drugs; moreover, it has been possible to see statistically significant differences between the “Gut” and “Gut/Serum” subsets too. We describe these general patterns in what follow, splitting the “Gut” set into its two very different subsets, “Gut-FL” and “Gut-noFL”.

The “Gut-FL” subset of “Gut” is clearly different from both drugs and “Gut/Serum” (and “Gut-noFL”) compounds: they are big, lipophilic, and flexible molecules, essentially devoid of scaffolds and with high hba, with very high structural homogeneity, and mostly neutral with a reduced share of acid molecules. They are, as expected by Lipinski’s and Veber’s rules, molecules unable to cross the gut wall.

As regarding shared properties between “Gut-noFL” and “Gut/Serum” that differentiate them from the “DrugBank” set, we can say that both gut metabolites subsets are characterized by larger proportions of “Organic acids and derivatives” and “Organic oxygen compounds”; less scaffolded (more linear) molecules; smaller and less aromatic scaffolds; almost no basic molecules, and with an increased proportion of zwitterions; and with significantly reduced logp, mw, hba, qed, nring, and naring, and higher hbd and fsp3.

In turn, the patterns that differentiate the “Gut/Serum” set from the “Gut-noFL” one are distribution of chemical classes and Tanimoto similarity closer to “DrugBank”; more aromatic and heterocyclic scaffolds; acid is the most frequent ionization class (neutral is in “Gut-noFL”); and with significantly lower rb, fps3, and higher hdb, hba, nring, naring.

Some of these differential patterns are reflected at the level of chemical classes: acidic “Benzenoids” are significantly enriched in “Gut/Serum”, while neutral ones are in “Gut-noFL”; acid and zwitterionic “Organic acids and derivatives” are enriched in “Gut-noFL”, while only zwitterions are in “Gut/Serum”; neutral “Steroids and steroid derivatives” are enriched in “Gut/Serum”, while in “Gut-noFL” the enriched ionization class is the acid one; etc.

In addition to these patterns, we have developed a novel Super Learner model to predict gut permanence. Super Learners [35] are a recent approach for stacking multiple Machine Learning models, that asymptotically improves or at least performs as well as any of the base models without overfitting, since the predictive variables of the meta-model are out-of-fold predictions of the base models. In this way, they automatically build an optimal weighted combination of candidate or base learners that minimize the generalization error rate [35, 68]. Although the use of Machine Learning in modeling quantitative structure–activity relationships (QSAR) is an area with decades of experience [69–74], due to the typical non-linearity and complexity of the associations with countless predictive variables, the use of SuperLearners or other approaches for model stacking is relatively scarce in the field, with just a few examples in the literature [75, 76]. This could be due to the relative newness of the SuperLearner method [35, 68], coupled with the recent spate of Deep Learning methods that have absorbed most of the efforts in this field [69, 77–79]. In our case, the use of graph Deep Learning models, alone or concatenated with fully connected networks, worsened external prediction compared to the SuperLearner, which could probably be due to the reduced size of the dataset, as Deep Learning models are more appropriate for “Big Data” sets.

The model for gut permanence here described clearly outperforms typical rule-based predictive approaches for oral absorption, like Lipinksi’s or Veber’s, mainly because of their inability to predict the “Gut-noFL” subset of “Gut Lingerers”. This new tool can aid in the development of drugs based on gut metabolites in order to predict gut permanence for new molecules. It can also be used in metabolome research, to predict the compartments where putative new metabolites could be found. The model can be downloaded at https://github.com/bbu-imdea/gutmetabos.

On the other hand, the approach for gut-targeted drug design assumed in this analysis and model is based on the molecular structure of the drug, which is alternative and could be complementary of other approaches based on drug delivery [25–27]. In our case, the permanence or not of the molecule in the gut would be due to the intrinsic capacity of the molecule to engage into a particular combination of paracellular or transcellular diffusion, as well as active or passive mediated transport, instead of specialized drug delivery systems. Our dataset is phenomenological in nature and does not allow us to ascertain the mechanism underlying gut permanence, but the patterns observed and the SuperLearner model allow the prediction of gut permanence from molecular structure. In this way, this approach is similar to Lipinsky or Veber’s rules, that is, based on medicinal chemistry, although with a different endpoint.

We acknowledge some possible imperfections in our dataset, as the collection of gut metabolites is based on multiple samples that can be obtained with different depths and with different backgrounds, and it is possible that for example, some compound of low but not null bioavailability, that in principle would be with more probability in the gut set, has by chance been detected in both the gut and the serum set, or even only in the later. Alternatively, it is possible that some highly bioavailable compound has only been detected in the gut set. Moreover, in some cases, detecting a compound in serum could be due to de novo synthesis in that compartment, and not to gut wall crossing. We think, however, that these chance compartment swaps or misassignments would correspond, if present, to a minimal proportion of compounds that otherwise would not change the qualitative and quantitative conclusions of this work, given the large number of compounds of the sets.

The thorough analyses of patterns and predictive model for gut metabolites here described can illuminate the rational design of gut targeted drugs taping from the microbiome research. However, the actual generation of such a drug is a complicated process that must address additional issues: target engagement (especially for intracellular targets), solubility, chemical stability, etc. In the case of drugs remaining in the gut, in principle there would be reduced toxicity and distribution issues, but additional complications can appear. For example, a metabolite locally produced in the gut, if administered orally could potentially be absorbed in the upper digestive tract, or be degraded in the stomach, and this previously unknown fact could affect molecules derived from it too, thus precluding oral administration. All in all, we expect that the current work will speed up the generation of the first successful examples of this exciting new drug modality.

### Supplementary Information

**Additional file 1: Table S1.** Prediction statistics for gut permanence prediction SuperLearner using cluster-based train/test splits. **Figure S1.** Distribution of ionization states across the four compound sets: DrugBank, and gut metabolites sets at pH = 6.0.** Figure S2.** Distribution of ionization states across the four compound sets: DrugBank, and gut metabolites sets at pH = 6.4. **Figure S3.** Distribution of ionization states across the four compound sets: DrugBank, and gut metabolites sets at pH = 7.0

## Data Availability

The dataset used and the Super Learner with instructions for training and executing are freely available in https://github.com/bbu-imdea/gutmetabos.
