# Supplementary material for: Analysis of metabolites in human gut: illuminating the design of gut-targeted drugs
Source: J Cheminform. 2023 Oct 13;15:96. doi: 10.1186/s13321-023-00768-y (PMC10571276; doi:10.1186/s13321-023-00768-y)
Supplement: Supplementary file 1 — Additional file 1: Table S1. Prediction statistics for gut permanence prediction SuperLearner using cluster-based train/test splits. Figure S1. Distribution of ionization states across the four compound sets: DrugBank, and gut metabolites sets at pH = 6.0. Figure S2. Distribution of ionization states across the four compound sets: DrugBank, and gut metabolites sets at pH = 6.4. Figure S3. Distribution of ionization states across the four compound sets: DrugBank, and gut metabolites sets at pH = 7.0 [file 13321_2023_768_MOESM1_ESM.docx]

Additional file information

**Analysis of Metabolites in Human Gut: Illuminating the Design of Gut-Targeted Drugs**

Alberto Gil-Pichardo,^1†^ Andrés Sánchez-Ruiz,^1†^ and Gonzalo Colmenarejo^1*^

^1^Biostatistics and Bioinformatics Unit, IMDEA Food, CEI UAM+CSIC, E28049 Madrid, Spain

†These two authors contributed equally to this work

^*^Corresponding Author

e-mail: [gonzalo.colmenarejo@imdea.org](mailto:gonzalo.colmenarejo@imdea.org)

| **pred** | **acc** | **prec** | **rec** | **F1** | **AUROC** | **AUPRC** |
| --- | --- | --- | --- | --- | --- | --- |
| **ext test** | 0.915 | 0.952 | 0.868 | 0.908 | 0.975 | 0.978 |
| **ext test FL** | 1 | 1 | 1 | 1 | NA | 0.978 |
| **ext test noFL** | 0.876 | 0.839 | 0.636 | 0.723 | 0.931 | 0.855 |
| **ext test stand** | 0.938 | 0.919 | 0.818 | 0.862 | NA | 0.916 |

**Table S1** *Prediction statistics for gut permanence prediction SuperLearner using cluster-based train / test splits*


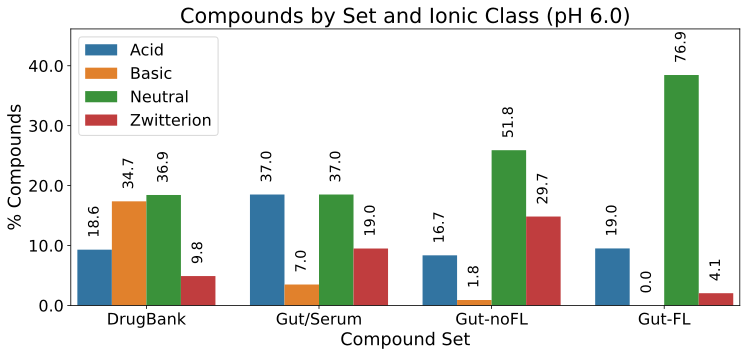


**Figure S1.** *Distribution of ionization states across the four compound sets: DrugBank, and gut metabolites sets at pH = 6.0*


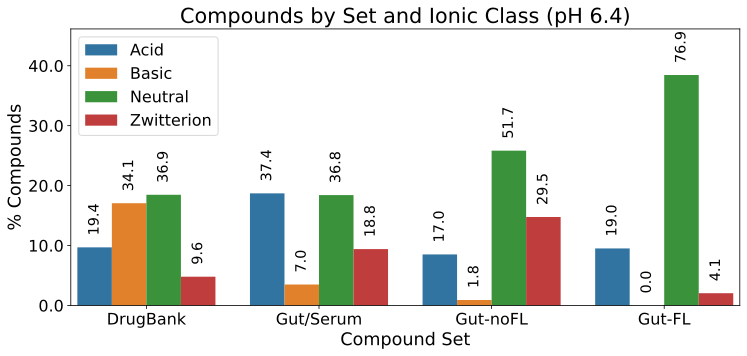


**Figure S2.** *Distribution of ionization states across the four compound sets: DrugBank, and gut metabolites sets at pH = 6.4*


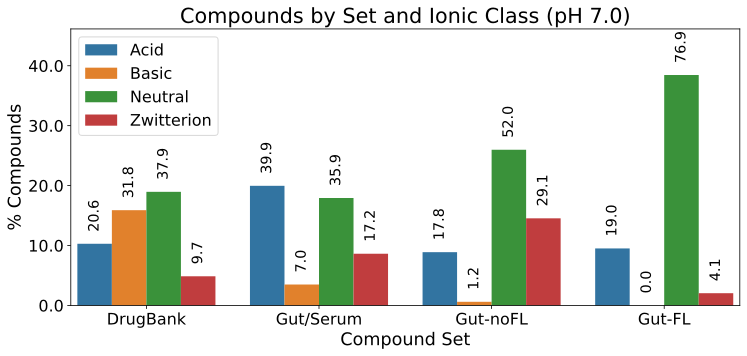


**Figure S3.** *Distribution of ionization states across the four compound sets: DrugBank, and gut metabolites sets at pH = 7.0*
